# Distinct cerebrovascular features in patients with ADA2 deficiency

**DOI:** 10.1186/1546-0096-13-S1-P157

**Published:** 2015-09-28

**Authors:** MS Severino, R Caorsi, C Gandolfo, C Martinetti, A Martini, M Gattorno

**Affiliations:** 1 G. Gaslini Institute, Department of Neuroradiology, Genova, Italy; 2 G. Gaslini Institute, 2nd Division of Pediatrics, Genova, Italy; 3 University of Genova, Department of Pediatrics, Genova, Italy

## Background

Mutations of CECR1 have been recently reported as causative of an inflammatory condition characterized by an early onset vasculopathy resembling polyarteritis nodosa. The clinical manifestations of the disease are heterogeneous with a wide range of severity. Patients with a more serve phenotype present early onset cerebral stroke, which can be either ischemic or hemorrhagic.

## Objectives

To describe the neuroradiologic features of patients affected by ADA2 deficiency with central nervous system (CNS) involvement.

## Methods

We reviewed the contrast-enhanced brain MR, MR angiography (MRA), and digital subtraction angiography (DSA) examinations of 3 male patients with a confirmed molecular diagnosis of ADA2 deficiency and CNS involvement: two brothers (R312X and E328D mutations in compound heterozygosis) and a third unrelated patient (T360A homozygosis). Age at first MR examination was 6 years, 1 year 5 months, and 6 years 2 months, respectively.

## Results

All patients presented multiple acute and/or chronic small ischemic infarcts involving the basal ganglia and the midbrain, in keeping with small-vessel occlusions (lacunar strokes). One patient additionally presented a large hemorrhagic infarct in the temporal lobe. Areas of focal accumulation of hemosiderin due to intraparenchymal bleeding were also noted. Interestingly, two patients presented an abnormal contrast-enhancing soft tissue in the interpeduncular cistern, encasing the midbrain perforating arteries. These neuroradiological findings regressed after the treatment with anti-TNF agents. The brain MRA and DSA showed no relevant vascular stenosis of the major arteries of the circle of Willis.

## Conclusions

Typical lacunar strokes in patients with ADA2 deficiency are due to occlusion of small perforating arteries, which can be missed on conventional arterial imaging focusing on the vessel lumen of relatively large arteries. We hypothesize that the abnormal soft tissue detected in the interpeduncular cistern in the present patients may represent an abnormal inflammatory perivascular response, leading to small vessel stenosis. Wider use of contrast-enhanced high-resolution MR examinations may better demonstrate inflammatory vascular manifestations in patients with ADA2 deficiency.

